# Mechanisms and therapeutic effectiveness of pulsed electromagnetic field therapy in oncology

**DOI:** 10.1002/cam4.861

**Published:** 2016-10-17

**Authors:** Maria Vadalà, Julio Cesar Morales‐Medina, Annamaria Vallelunga, Beniamino Palmieri, Carmen Laurino, Tommaso Iannitti

**Affiliations:** ^1^ Department of General Surgery and Surgical SpecialtiesSurgical ClinicUniversity of Modena and Reggio Emilia Medical SchoolModenaItaly; ^2^ Centro de Investigación en Reproducción AnimalCINVESTAV‐ Universidad Autónoma de TlaxcalaTlaxcalaMexico; ^3^ Department of Medicine and SurgeryCentre for Neurodegenerative Diseases (CEMAND)University of SalernoSalernoItaly; ^4^ Department of NeuroscienceSheffield Institute for Translational Neuroscience (SITraN)University of SheffieldSheffieldUnited Kingdom

**Keywords:** Cancer, electromagnetic therapy, oncology, pulsed electromagnetic fields, tumor‐specific frequencies

## Abstract

Cancer is one of the most common causes of death worldwide. Available treatments are associated with numerous side effects and only a low percentage of patients achieve complete remission. Therefore, there is a strong need for new therapeutic strategies. In this regard, pulsed electromagnetic field (PEMF) therapy presents several potential advantages including non‐invasiveness, safety, lack of toxicity for non‐cancerous cells, and the possibility of being combined with other available therapies. Indeed, PEMF stimulation has already been used in the context of various cancer types including skin, breast, prostate, hepatocellular, lung, ovarian, pancreatic, bladder, thyroid, and colon cancer in vitro and in vivo. At present, only limited application of PEMF in cancer has been documented in humans. In this article, we review the experimental and clinical evidence of PEMF therapy discussing future perspectives in its use in oncology.

## Introduction

Cancer is one of the most common causes of death worldwide and accounted for 8.2 million deaths in 2012 1. The number of cancer‐related deaths is predicted to increase to over 11 million by 2030 2. The types of cancer with the highest incidence are lung (1.59 million people), liver (745,000), stomach (723,000), colon and rectum (694,000), breast (521,000), and esophagus (400,000) 1. In oncology, the selection of correct treatment strategy, in early disease stages, is crucial to increase the probability of remission and improve survival. Available cancer treatments include chemotherapy, immunotherapy or antibody‐based therapy, radiation therapy, and surgery 3. The therapeutic strategy is chosen taking into account the individual patient's medical assessment, type of cancer, location, and disease stage 4. Multimodal treatments are often required to reduce the therapy‐induced side effects 5 related to pharmacological as well as other approaches including surgery 6. Chemotherapy‐induced side effects depend on various variables such as the drug employed, its dosage, and treatment duration. These side effects include pain, fatigue, throat and mouth sores, diarrhea, nausea, vomiting, constipation, and blood disorders. Side effects affecting the nervous system are commonly experienced with chemotherapy and include cognitive dysfunction, headache, dizziness, vision loss and vision disturbances such as blurred or double vision, changes in learning and memory, sexual dysfunction, ataxia, and peripheral neuropathy 7, 8, 9, 10, 11. Rashes, fever, hypotension, colitis or other gastrointestinal problems, and thyroid dysfunctions are immunotherapy‐related side effects 12. The main radiotherapy‐induced side effects are dry mouth and gum sores, jaw stiffness, nausea, lymphedema, swallowing difficulties, shortness of breath, breast or nipple soreness, rectal bleeding, incontinence, bladder irritation, and pituitary dysfunction 13. Surgical techniques, such as minimally invasive surgery, also result in pain, fatigue, appetite loss, swelling and bruising around the site of surgery, bleeding, infection, lymphedema, and organ dysfunction 14. Numerous studies support the development of new treatments in oncology to be added to the traditional protocols to increase the effectiveness of available treatments, reducing side effect profile, and the patients' quality of life 15, 16, 17, 18. Such resources include traditional Chinese medicine, Ayurvedic medicine, homeopathy, and naturopathy 19. While complementary and alternative medicine (CAM) is not generally considered part of conventional medicine, it has been widely used in the oncology field as an add‐on therapy to control patients' symptoms and improve their quality of life 20, 21, 22, 23, 24, 25, 26. The beginning of the 20th century saw the first therapeutic applications of CAM therapies for cancer treatment; these therapies include acupuncture, chromotherapy, therapeutic touch (reiki), and pulsed electromagnetic field (PEMF) therapy 4, 15, 27, 28, 29, 30. In this review, we have focused on PEMF therapy, a noninvasive technique characterized by electromagnetic fields inducing microcurrents to the entire body or locally to target specific body tissues. Exposure to PEMFs in the 0–300 Hz range is a therapeutic tool extensively used for the treatment of several pathologies including osteoarthritis, Parkinson's disease, postsurgical pain and edema, treatment of chronic wounds, and facilitation of vasodilatation and angiogenesis producing direct stimulation to excitable cells including nerve and muscle cells 31, 32, 33, 34. Stimulation with sufficient intensity and duration induces a current across targeted cell membranes, activating nerve cells or muscles to propagate action potentials 35, 36, 37. Indeed, PEMF therapy can be used as an adjuvant treatment to chemotherapy and radiotherapy with the aim of reducing their dosage, mitigating any harmful secondary side effects, and enhancing patient's prognosis 15, 35, 38, 39, 40.

### Aim and searching criteria

We reviewed in vitro, in vivo, and clinical studies employing PEMF therapy for cancer treatment published between 1976 and 2016. We searched Pubmed/Medline, Embase, Web of Science and Scopus using the keywords “PEMFs”, “cancer”, “magnet therapy”, “tumour specific frequencies” and “oncology” alone or combined. This review aims at describing the state of the art of PEMF therapy, discussing current understanding of the underlying mechanisms and outlining future therapeutic perspectives in oncology.

## In Vitro Studies

PEMF therapy has been extensively studied in vitro using various human cancer cell lines, such as pheochromocytoma‐derived (PC12), breast cancer (e.g., MCF7, MDA‐MB‐231 and T47D), and colon cancer (SW‐480 and HCT‐116) 41, 42, 43, 44, 45. These studies have shown that PEMF therapy may exert proliferative inhibition and mitotic spindle disruption 18, 40, block the development of neovascularization required for tumor supply 46, 47, 48 and exacerbate an inherent or induced genetic instability by reducing the stringency of the late‐cycle (G2) checkpoint 49. While chemotherapy is not specific to cancer cells and targets all rapidly dividing cells 50, 51, 52, PEMFs exert selective cytotoxic effect on neoplastic cells 15, 40, 53, 54, 55 making this therapy a highly promising strategy.

In the next subparagraphs, we will review studies employing PEMF therapy in different cell lines as a model to study specific types of cancer (Table 1).

**Table 1:** In vitro studies of PEMF therapy in oncology

Author(s), year	Cell type	Treatment	Main findings	References
Crocetti et al., 2013	Human breast adenocarcinoma cells (MCF7) and nontumorigenic cells (MCF10)	Daily 60‐min PEMF therapy session (20 Hz; 3 mT) for 3 days	PEMFs increased apoptosis in MCF7 cells but had no effect on MCF10 cells	38
Filipovic et al., 2014	Human breast cancer (MDA‐MB‐231) and colon cancer (SW‐480 and HCT‐116) cell lines	24 and 72 h exposure to PEMF therapy (50 Hz; 10 mT)	PEMFs increased apoptosis in MDA‐MB‐231 (55% and 20%), SW480 (11% and 6%), and HCT‐116 cell lines (2% and 3%) after 24 and 72 h exposure, respectively, compared with untreated control cancer cell lines	58
Morabito et al., 2010	Undifferentiated PC12 pheochromocytoma cells and differentiated PC12 cells	Short PEMF therapy session (50 Hz, 0.1–1.0 mT) for 30 min, and long‐term PEMF session (50 Hz, 0.1–1.0 mT) for 7 days	30‐min PEMF session in undifferentiated PC12 cells increased ROS levels and decreased catalase activity. No change in intracellular Ca^2+^ concentration was observed. 7‐day PEMF therapy session in undifferentiated PC12 cells resulted in increased intracellular Ca^2+^ concentration and increased catalase activity. No significant findings were observed in differentiated PC12 cells	41

PEMF, pulsed electromagnetic field; ROS, reactive oxygen species.

### Studies of PEMF therapy in human breast cancer and colon cancer cell lines

A study by Crocetti and coworkers 38 investigated whether ultra‐low intensity and frequency PEMF therapy could induce apoptosis in human breast adenocarcinoma cells (MCF7). PEMF exposure was cytotoxic to MCF7 cells, but not to normal breast epithelial cells (MCF10). Both MCF7 and MCF10 cells were exposed to PEMF therapy and the cytotoxic indices were measured in order to design PEMF paradigms that could reduce selectively neoplastic cell proliferation. The PEMF parameters tested were: (1) frequency of 20 Hz, (2) intensity of 3 mT and (3) exposure time of 60 min/day for up to 3 days. Four independent methods of monitoring cancer‐induced apoptosis (trypan blue assay, apoptosis determination by DNA strand break detection, analysis of cellular electrical properties by means of impedance microflow cytometer, and apoptosis determination by Annexin V staining) showed that this specific set of PEMF parameters was cytotoxic to breast cancer cells. While this treatment selectively induced apoptosis of MCF7 cells, it had no effect on MCF10 cells that were more resistant to apoptosis in response to PEMFs. Although these results are encouraging, PEMF exposure was limited to 3 days. Long‐term PEMF exposure needs to be assessed in further studies based on the concept that PEMF effectiveness is strictly linked to the signal parameters, exposure magnitude, duration, signal shape, duration of treatment as well as the type of cells exposed to the magnetic field 56, 57.

The antineoplastic effect of PEMFs has also been investigated in human breast cancer MDA‐MB‐231, colon cancer SW‐480, and HCT‐116 cell lines. These cells were exposed to 50 Hz PEMFs for 24 and 72 h 58. PEMFs decreased the number of viable cells in all the cell lines tested, reaching 55% after 24 h and 20% after 72 h in the MDA‐MB‐231 cell line, 11% after 24 h and 6% after 72 h in the SW480 cell line, and 2% after 24 h and 3% after 72 h in the HCT‐116 cell line, compared with unexposed cancer cell lines used as controls, as assessed by a computer reaction‐diffusion model, a mathematical model widely employed to study cell proliferation and infiltration 59. The lower percentage inhibition of neoplastic cell proliferation was observed after 72 h, showing that PEMF therapy had antiproliferative activity which decreased over time. This action is exerted in vitro by interfering with microtubule spindle polymerization. Indeed, PEMF exposure reduces the fraction of polymerized microtubules, disrupts the mitotic spindle structure, inhibits cell division, thereby leading to chromosome mis‐segregation and cancer‐induced apoptosis 60. In summary, studies in human breast and colon cancer cell lines are promising and warrant further investigations.

### Studies of PEMF therapy in pheochromocytoma‐derived cells

PEMF signal parameters have been extensively utilized on diverse cell types to determine in vitro effectiveness 61, 62. For example, Morabito and coworkers 41 investigated cell responsiveness and in vitro neuritogenesis following PEMF exposure. They specifically focused on PEMF ability to modify morphology, proliferation, and differentiation in PC12 pheochromocytoma cells. Furthermore, they assessed whether PEMFs can induce variable and species‐specific alterations in the oxidative stress pathway such as Ca^2+^‐dependent oxidative stress which enhances free radical production, particularly via the Fenton reaction, leading to apoptotic cell death 63, 64, 65, 66, 67, 68, 69. Undifferentiated and differentiated [supplemented with 50 ng/mL of nerve growth factor (NGF)] PC12 cells were exposed to 50 Hz PEMF therapy (0.1–1.0 mT), and cell growth and viability were evaluated after immediate (30 min) or long‐term exposure (7 days), using colorimetric and morphological assays. The long‐lasting exposure to PEMFs did not affect the biological response in terms of proliferation and neuritogenesis. Thirty‐minute PEMF exposure at 1.0 mT in undifferentiated PC12 cells increased the levels of reactive oxygen species (ROS) and decreased catalase activity, an indicator of oxidative stress. Conversely, long‐term PEMF exposure of undifferentiated PC12 cells also increased catalase activity that could reflect the absence of ROS accumulation and a possible adaptation cell response to PEMFs. During immediate PEMF exposure in undifferentiated PC12 cells, no change in intracellular Ca^2+^ concentration was observed, while it increased after long‐term exposure. This enhanced calcium level could activate, through voltage‐gated (L‐type) calcium channels, signaling pathways and lead to the expression of genes modulating cell differentiation, survival, and apoptosis such as extracellular signal‐regulated kinases, c‐Jun N‐terminal protein kinase/stress‐activated protein kinase, and p38 70, 71, 72, 73. In particular, the undifferentiated PC12 cells were more sensitive to PEMFs exposure, while the differentiated PC12 cells were more stable and resistant to stress, probably due to the action of the cell surface NGF receptors such as p75NR 74.

Further studies are necessary to identify the ROS/intracellular Ca^2+^ cross‐talking pathway activated by PEMF therapy. However, the study by Morabito and coworkers supports the hypothesis that ROS and Ca^2+^ could be the cellular “primum movens” of PEMF therapy‐induced effects, as observed in pheochromocytoma cells.

## In Vivo Studies

Several studies investigated the antineoplastic effect of PEMFs using widely employed animal models of several types of cancer, including breast cancer, hepatocellular carcinoma (HCC), and melanoma (Table 2) 4, 48, 75, 76, 77, 78.

**Table 2:** In vivo studies of PEMF therapy in oncology

Author(s), year	Animal model (number of animals, study design)	Route of administration	Treatment	Main findings	References
Tatarov et al., 2011	12 T‐cell‐immunodeficient Swiss outbred female nude mice (Cr:NIH(S)‐*nu/nu*), divided into 4 groups (*n* = 3 each)	Orthotopic injection of metastatic mouse breast tumor cell line [EpH4‐MEK Bcl2^13^ cells (1 × 10^6^)] into the mammary fat pad	Group 1, 2 and 3 were exposed to PEMFs (1 Hz, 100 mT) daily for 60, 180, or 360 min, respectively, for 4 weeks; group 4 did not receive any treatment and was used as control	Mice exposed for 60 and 180 min daily showed a 30% and 70% tumor reduction, respectively, at week 4, if compared to baseline	85
Emara et al., 2013	60 rats (strain not reported) divided into 6 groups	Intraperitoneal administration of a carcinogenic agent, DEN	Group 1 (naive rats) received PEMF therapy (2‐3 Hz, 0.004 T) for 30 min/day for 6 days/week for 4 weeks; group 2 (naive rats) received PEMF therapy (<1 Hz, 0.6 T) 15 min/day for 6 days/week for 4 weeks; group 3 (naive rats) was left untreated; group 4 (HCC rats) received PEMF therapy (2‐3 Hz, 0.004 T) for 30 min/day for 6 days/week for 4 weeks; group 5 (HCC rats) received PEMF therapy (<1 Hz, 0.6 T) 15 min/day for 6 days/week for 4 weeks; group 6 (HCC rats) was left untreated.	A significant decrease in serum AFP level and a slight improvement in dielectric properties of liver tissues was observed in HCC rats treated with PEMFs. These results were confirmed by electron microscopy and histological analysis showing HCC regression. No changes in histopathology and dielectric properties of liver tissue were observed in naive rats exposed to PEMFs.	91
Nuccitelli et al., 2006	23 SKH‐1 immunocompetent, hairless, albino mice	Single subcutaneous injection of B16 murine melanoma cells (1 × 10^5^) on the dorsal side of the mouse ear	30‐min PEMF therapy session (0.5 Hz, 0.2 T) three times a day for 6 days	All mice exhibited significant pyknosis, shrinkage of the tumor cell nuclei by 54% within a few minutes after PEMF therapy and by 68% within 3 h and reduction in the blood flow in about 15 min following PEMF therapy	95
Nuccitelli et al., 2010	Four female immunodeficient, hairless, albino Nu/Nu mice	Single subcutaneous injection of murine melanoma cells (B16‐F10‐eGFP, 1 × 10^5^) on the mouse skin	Daily 6‐min PEMF session (5–7 Hz, 0.2 T) for 10 days	Melanoma cells shrank within an hour post PEMF therapy, exhibiting pyknosis within 24 h post treatment. PEMFs‐treated mice showed complete remission of melanoma	100

PEMF, pulsed electromagnetic field; DEN, *N*‐diethylnitrosamine; AFP, alpha‐fetoprotein; HCC, hepatocellular carcinoma

### PEMF therapy effectiveness in mouse models of breast cancer

PEMF therapy effectiveness on tumor growth and viability has been tested in mouse models of breast cancer. For example, xenograft mouse models are widely used to study breast cancer. This model is obtained by injection of human breast cancer cells including estrogen‐negative (MDA‐MB‐231) and estrogen‐positive (MCF7) breast carcinoma cell lines or mouse breast cancer cells including EpH4 mammary epithelial cells or mitogen‐activated protein kinase (MEK)‐transformed EpH4 cells subcutaneously, intravenously, intracardially, or orthotopically, four times every 5 days, into the mammary fat pad of immunocompromised mice 79, 80. The injected cells are highly invasive in vitro and tumorigenic when transplanted into the mammary fat pad. After a week from the last injection, the mouse is palpated biweekly for mammary tumors and the dimensions of tumors are measured using an external caliper daily. Mice are euthanized when the tumor size becomes ulcerated with macro‐metastases, mainly in liver, bone, and brain 81, 82, 83, 84. For example, EpH4‐MEK Bcl2^13^ cells (1 × 10^6^) transfected with a luciferase expression vector (p*β*P2‐PolII‐luciferase) were injected into the mammary fat pad in 12 T‐cell‐immunodeficient Swiss outbred female nude mice (Cr:NIH(S)‐*nu/nu*) 85. Mice were divided into four groups (*n* = 3 each). Group 1, 2, and 3 were exposed to PEMF therapy (1 Hz, 100 mT) daily for 60, 180, or 360 min, respectively, for 4 weeks, while group 4 did not receive PEMF therapy and was used as control. All mice were monitored for tumor growth by body bioluminescence imaging once every 2 to 4 days for 4 weeks. Then, all the mice were sacrificed and skin, liver, lung, and spleen samples were collected for histopathologic analysis. Mice exposed to PEMFs for 60 and 180 min daily showed a 30% and 70% breast tumor reduction, respectively, at week 4, if compared to baseline. Mice exposed to PEMFs for 360 min daily, showed a suppression of tumor growth at week 4. In summary, this study shows that the time of PEMF exposure is critical to determine its effectiveness. Mice exposed for longer duration (360 min daily for 4 weeks) showed a significant reduction in tumor size, due probabily to the inhibition of angiogenesis that may suppress the formation of blood vessels in tumor tissues, reducing the tumor growth.

### Antineoplastic effect of PEMF therapy in rodent models of hepatocellular carcinoma

Chemically induced HCC is a widely used model of hepatocarcinogenesis that mimics the development of fibrosis and cirrhosis. This model is obtained by intraperitoneal administration of a carcinogenic agent, *N*‐diethylnitrosamine (DEN; 50–100 mg/kg mouse body weight) alone or followed by oral administration of a nongenotoxic liver tumor promoter [phenobarbital (PB)]. DEN induces damage to DNA, proteins, and lipids, leading to hepatocyte death 86. It is hydroxylated to *α*‐hydroxylnitrosamine, mediated by cytochrome P450 enzymes which are primarily located in the centrilobural hepatocytes. Then, an electrophilic ethyldiazonium ion is formed and causes DNA damage by reacting with nucleophiles. Three to four weeks following the last injection, mice receive drinking water containing PB (0.07%) that increases the expression of cytochrome P450, inducing oxidative stress and resulting in HCC development after 6 months from PB administration 86, 87, 88, 89, 90. Emara and coworkers evaluated the safety and effectiveness of PEMFs with different intensity and frequency in a rat model of DEN‐induced HCC (75 mg/kg body weight, once a week for 3 weeks) 91. Sixty rats were divided into six groups: Group 1 (naive rats) received PEMF therapy (2‐3 Hz, 0.004 T) for 30 min/day for 6 days/week for 4 weeks; group 2 (naive rats) received PEMF therapy (<1 Hz, 0.6 T) 15 min/day for 6 days/week for 4 weeks; group 3 (naive rats) was left untreated; group 4 (HCC rats) received PEMF therapy (2‐3 Hz, 0.004 T) for 30 min/day for 6 days/week for 4 weeks; group 5 (HCC rats) received PEMF therapy (<1 Hz, 0.6 T) 15 min/day for 6 days/week for 4 weeks; group 6 (HCC rats) was left untreated. No changes in histopathology and dielectric properties of liver tissue were observed in naive rats exposed to PEMFs supporting its safety. In HCC rats exposed to PEMFs, a significant decrease in AFP level (AFP is a serum glycoprotein often elevated in HCC patients and used as a carcinoma marker in the clinic) was reported together with a slight improvement in dielectric properties of liver tissue. These results were confirmed by electron microscopy and histological analysis showing HCC regression. Altogether this evidence supports the antineoplastic activity of PEMF therapy in the rat model of DEN‐induced HCC and warrants further investigations.

### PEMF therapy effectiveness in murine melanoma models

The most frequently used murine melanoma model is the syngeneic B16 model. It is obtained by a single subcutaneous injection of 1 × 10^5^ B16 murine melanoma cells on the dorsal side of the mouse ear. Melanoma nodules 5–6 mm in diameter develop 7 days post‐injection 92, 93, 94. The melanoma model in SKH‐1 hairless mice has been used to investigate the effectiveness of PEMF therapy (0.5 Hz, 0.2 T, 30 min/day). Mice (*n* = 23) received 1–3 PEMF treatments daily for 6 days and were monitored for tumor growth, daily, by optical methods, such as transillumination and power Doppler ultrasound reconstructions that display blood flow images for each tumor 95. Then, all the mice were sacrificed and skin tissues were collected for histopathological analysis. All mice exposed to PEMFs exhibited significant pyknosis, shrinkage of the tumor cell nuclei by 54% within a few minutes after PEMF therapy and by 68% within 3 h and reduction in the blood flow in about 15 min following PEMF therapy. These effects may be due to PEMF therapy that stimulates murine melanoma to self‐destruct by triggering rapid pyknosis of tumor cell nuclei and reducing blood flow 96, 97, 98, 99. A further study 100 optimized the PEMF therapy parameters pulse number, amplitude, and frequency to completely suppress melanoma with a single treatment. In this study, four female immunodeficient, hairless, albino Nu/Nu mice received a single PEMF treatment for 6 min using the following parameters: 2.700 pulses, amplitude of 30 kV/cm and frequency of 5–7 Hz for 10 days. After 2–4 weeks, mice were sacrificed and skin samples were processed for histology. Melanoma cells shrank within an hour post PEMF therapy, exhibiting pyknosis within 24 h post PEMFs and showing a complete remission of melanoma in all the mice, as assessed by in vivo imaging (transillumination and photography). To evaluate the safety of PEMF therapy, the authors recorded the physiological parameters and introduced a miniature thermocouple into the tumor for simultaneous measurement of intratumoral temperature during PEMF treatment; body temperature and systolic blood pressure showed no significant changes, while the intratumoral temperature was ~6–7°C, evidencing that, by limiting the frequency to 7 Hz or less, it was possible to avoid heating the tumor to hyperthermia temperatures potentially leading to damage of the surrounding tissues. Evidence of efficacy of a single PEMF treatment on mouse skin cancer resulting in suppression of tumor growth and induction of apoptosis is promising for translational applications.

## Clinical Studies

The use of PEMF therapy in oncology is still limited (Table 3) 4. The first study utilizing PEMF therapy was conducted by Barbault and coworkers who hypothesized that a combination of specific frequencies, defined tumor‐specific frequencies, may display therapeutic effectiveness for localized treatment of tumors 15. They identified a total of 1524 tumor‐specific frequencies, ranging from 0.1 to 114 kHz, consisting in the measurement of variations in skin electrical resistance, pulse amplitude, and blood pressure in 163 patients affected by different types of cancer including brain tumors, colorectal cancer, HCC carcinoma, pancreatic, colorectal, ovarian, breast, prostate, lung, thyroid, and bladder cancer and exposed to the radiofrequency system. Self‐administered PEMF therapy for 60 min, three times a day, for an average of 278.4 months was offered to only 28 patients with advanced cancer (breast cancer [*n* = 7], ovarian cancer [*n* = 5], pancreatic cancer [*n* = 3], colorectal cancer [*n* = 2], prostate cancer [*n* = 2], glioblastoma multiforme [*n* = 1], HCC carcinoma [*n* = 1], mesothelioma [*n* = 1], neuroendocrine tumor [*n* = 1], non‐small‐cell lung cancer [*n* = 1], oligodendroglioma [*n* = 1], small‐cell lung cancer [*n* = 1], sarcoma [*n* = 1] and thyroid tumor [*n* = 1]). None of the patients who received PEMF therapy reported any side effects; four patients presented stable disease for 3 years (thyroid cancer with biopsy‐proven lung metastases), 6 months (mesothelioma metastatic to the abdomen), 5 months (non‐small‐cell lung cancer), and 4 months (pancreatic cancer with biopsy‐proven liver metastases), respectively.

**Table 3:** Clinical studies of PEMF therapy in oncology

Author(s), year	Study design	Number of patients	Pathology	Treatment	Outcomes	Side effects	References
Barbault et al., 2009	Compassionate and investigational clinical trial	28	Glioblastoma multiforme, mesothelioma, oligodendroglioma, sarcoma, HCC and breast, colorectal, lung, neuroendocrine, ovarian, pancreatic, prostate and thyroid cancers	60‐min PEMF session (0.1 Hz–114 kHz, 1.5 T) three times a day for 278.4 months	One patient with thyroid cancer, one patient with mesothelioma metastatic to the abdomen, one patient with non‐small‐cell lung cancer and one patient with pancreatic cancer with biopsy‐proven liver metastases presented stable disease for 3 years, 6 months, 5 months and 4 months, respectively	None reported	15
Costa et al., 2007	A single‐group, open‐label, phase I/II clinical trial	41	Advanced HCC	Daily 60‐min PEMF session (100 Hz–21 kHz, 1.5 T) three times a day for 6 months	Five patients reported complete disappearance and two patients reported decrease in pain shortly after treatment. Four patients showed a partial response to treatment, while 16 patients had stable disease for more than 12 weeks	None reported	102

PEMF, pulsed electromagnetic field; HCC, hepatocellular carcinoma.

PEMF therapy has also been employed for the treatment of HCC. Therapies for this disease are needed, especially for patients at an advanced disease stage who cannot tolerate chemotherapy or intrahepatic interventions because of impaired liver function 101. The feasibility of PEMF therapy for treatment of HCC has also been investigated in a single‐group, open‐label, phase I/II clinical study 102. Forty‐one patients with advanced HCC received very low levels of PEMFs modulated at HCC‐specific frequencies (100 Hz–21 kHz) and received three‐daily 60 min outpatient treatments. No adverse reactions were observed during PEMF treatment. Five patients reported complete disappearance and two patients reported decrease in pain shortly after beginning of treatment. Four patients showed a partial response to treatment, while 16 patients (39%) had stable disease for more than 12 weeks. This study shows that PEMF therapy provides a safe and well‐tolerated treatment, as well as evidence of antineoplastic effects in patients with HCC.

In summary, encouraging findings warrant randomized clinical studies to determine the effectiveness of amplitude‐modulated PEMF therapy that can delay cancer progression and increase overall survival in patients. The increased knowledge of tumor‐specific frequencies and the preliminary evidence that additional tumor‐specific frequencies may yield a therapeutic benefit provide a strong rationale for the novel concept that administration of a large number of these frequencies may result in successful long‐term disease management.

## Discussion and Conclusions

In vitro studies support antineoplastic and antiangiogenic effects of PEMF therapy. Several mechanisms of PEMF therapy have been elucidated. For example, PEMFs inhibit cancer growth by disrupting the mitotic spindle in a process mediated by interference of spindle tubulin orientation and induction of dielectrophoresis. Furthermore, PEMF therapy modulates gene expression and protein synthesis interacting with specific DNA sequences within gene promoter regions 18, 38, 40, 41, 58, 103. In addition, PEMFs inhibit angiogenesis in tumor tissues, suppressing tumor vascularization and reducing tumor growth, as shown by in vivo studies 95, 96, 97, 98, 99, 104.

The specific claim, supported by the described in vivo studies, is that all treated groups showed slower tumor growth rate if compared with untreated control group, confirming that PEMF therapy can modulate the physiology and electrochemistry of cancer cells and influence cell membrane systems and mitosis. In addition, PEMFs induce some changes in membrane transport capacity through impacting the osmotic potential, ionic valves and leading to reduction in cellular stress factors, increase in the rate of DNA transcription, and modulation of immune response 105.

PEMFs have also an immunomodulatory effect, as supported by in vivo evidence showing an increase in tumor necrosis factor alpha levels that induce an anti‐tumoral response, leading to the activation of a proapoptotic pathway induced by caspase‐8 interaction with Fas‐associated death domain, in the spleen of the murine melanoma mouse model after a 16‐day therapy 78. Changes in blood pressure, skin electrical resistance, and pulse amplitude in 163 oncology patients exposed to tumor‐specific PEMF frequencies have also been reported suggesting that PEMF therapy does not only target neoplastic cells, but may also have systemic effects 15. However, long‐term PEMF treatment in HCC patients is not toxic, confirming the safety of PEMF therapy that employs 100,000 times lower frequencies if compared with radiofrequency ablation that is also employed for treatment of HCC 55.

In conclusion, only two clinical studies have used PEMF therapy for cancer treatment. These studies show that PEMF therapy is safe and promising compared to other available cancer therapies. In the future, PEMFs could be used not only as primary therapy but also in combination with other common antineoplastic therapies. Given that new portable and affordable PEMF devices are increasingly available on the market, future controlled clinical studies are expected to further determine the potential of PEMF therapy in oncology.

## Conflict of Interest

The authors certify that there is no conflict of interest with any financial organization regarding the material discussed in the manuscript.
